# Copolymerization-Regulated Hydrogen Bonds: A New Routine for High-Strength Copolyamide 6/66 Fibers

**DOI:** 10.3390/polym14173517

**Published:** 2022-08-27

**Authors:** Zichao Wang, Ming Song, Xilin Li, Jizong Chen, Tiexian Liang, Xin Chen, Yurong Yan

**Affiliations:** 1 School of Material Science and Engineering, South China University of Technology, Guangzhou 510640, China; 2 Guangdong Xinhui Meida Nylon Co., Ltd., Jiangmen 529100, China; 3 Key Lab of Guangdong High Property & Functional Polymer Materials, Guangzhou 510640, China

**Keywords:** the molecular design of polymers, in-site FTIR, mechanical properties, bonded and free hydrogen bonds, two-dimensional spectroscopy analysis

## Abstract

Hydrogen bond interactions are important for nylon fibers, which improve its mechanical properties and crystallization behavior, while hindering the movement and orientation of the molecular chain during the drawn process. In this study, hexamethylene adipamide was used as the second monomer in copolymerization with ε-caprolactam to obtain copolyamide 6/66 (CoPA), and high-tenacity fibers with a maximum value up to 8.0 cN/dtex were achieved by a multi-step drawn and thermal setting process. Results show that the hexamethylene–adipamide ratio affected the draw ratio (DR) of the as-spun fiber, on the tenacity of final high-performance fiber, and on crystalline. Both DR and tenacity showed evident increases with the hexamethylene–adipamide ratio up to 6% in CoPA and then changed smoothly. However, XRD and DSC results illustrate a decreased tendency with regard to crystallinity. The attenuated in-site total reflection Fourier transform infrared (ATR-FTIR) spectra were used to study the hydrogen bond interaction between the C=O group and N–H group and the crystal form of the fiber. Results show that the copolymerization destroyed the regularity of the main chain of CoPA and reduces the interaction of interstrand hydrogen bonds, facilitating the formation of the γ-crystalline form in as-spun fibers, fulfilling the transition from the γ to α crystalline form during the fiber-drawing step because of the release of the C=O group and N–H group from the hydrogen bond interaction at an elevated temperature close to the molten temperature of CoPA, and then reforming during the thermal-setting step which soiled the crystalline and improved the tenacity of the fiber. The copolymerization with a homologous monomer regulates the hydrogen bond interaction, fulfills the high drawn ratio and high tenacity fiber, and provides a new route for high-performance fiber preparation using traditional fiber formation of polymers.

## 1. Introduction

Hydrogen bonds, a mainstream, non-covalent interaction, are important to materials containing functional groups such as C=O, OH, COOH, F, NH_3_, etc. [1]. It influences molecular conformation, regulates the intermolecular action, and thus helps stabilizing supramolecular structures in biopolymer systems [2], providing a high strength to composite paper [3], building the network of high-performance hydrogels [4], improving thermal conductivity in hybrid liquid-crystal systems [5], and fulfilling chemically responsive networks or building dynamic structures for smart materials [5,6]. Polyamide(PA), prepared from dicarboxylic acids and diamines (AABB type) or from ω-aminocarbonxylic acids (AB type), is one of the most common raw material fibers used for both textiles and plastics [7,8]. PAs are composed of sheets of fully extended planar chains characterized by a group of –CONH–. The interstrand and intrastrand hydrogen bonds of PAs dominate the regular arrangement of macromolecules within crystals [9]. The density of the hydrogen bond interaction also influences the thermal properties and mechanical properties. PA66 shows a higher melting point temperature, stronger mechanical properties, and more dynamic properties than those of PA6. The former can form a planar zig-zag chain structure, allowing full hydrogen bonds between the N–H and C=O groups of adjacent chains, while the latter can form a planar zig-zag structure, allowing only one-half of the available groups to be hydrogen–bonded [10]. 

Regular hydrogen bonds improve the liner arrangement of the molecular chain, and favors the stable crystal in a condensed state, strengthening the tenacity of the polyamide fiber and improving the melting point. However, hydrogen bond interaction ties the adjacent macromolecule too strongly to free the molecular chain during the drawn step in fiber or film processing [11,12].

Lots of research has been focused on how to destroy the hydrogen bond between adjacent chains during the drawn step in fiber formation, and then how to re-form it during the thermal-setting step. Using plasticizers or complexions, such as Iodine complexation [13], anhydrous ammonia, metal chloride [14], rare earth chloride, and ionic liquids [15], adjusts hydrogen bond interaction and increases the movement of the molecular chain, improving the drawn condition, but it also acts as impurities worsen the dyeability or even color the fiber matrix. Removing it from the polymer matrix is needed after spinning in case it reduces other properties [16]. Improved process units that achieve a high-tenacity polyamide or polyester fiber have been studied for decades, such as high-speed melt spinning [17], spin line heating (SLH) and spin line cooling (SLC) [18], high-temperature zone drawing by CO_2_ laser thinning [19], and drawing in a liquid isothermal bath [20] and horizontal isothermal bath [21]. With the help of the orientation of in-site graphene, polyamide filaments characterized by a tensile strength of 7.2 g/d was prepared [22]. In industry, through a specially designed drawn unit, polyamide 6(PA6), with a high tenacity of 7.23 cN/dtex, it is possible using low-relative-viscosity raw material [23]; as the relative viscosity of the raw material changes to a higher value, the highest tenacity up to 7.65 cN/dtex was achieved on multistep drawn equipment [24].

Is it possible to achieve high-tenacity polyamide 6(PA6) fibers by the molecular design of the polymer using the copolymerization method instead of using additives? Here, different aliphatic monomers are selected. ε-caprolactam is the raw material for polymerization of PA6 which is the most commonly used raw material for the synthesis fiber in the textile and tech-textile fields. Hexamethylene adipamide salt (HA salt) is the oligomer obtained by the reaction of adipic acid and hexamethylene diamine and is the raw material of polyamide 66 (PA66), which is also a typical raw material fiber for technical textiles. PA6/x (here x means the second comonomers other than ε-caprolactam) has been successfully manufactured in industry and has been used in transparent film or plastic, fishing monofilament, and hot melt adhesives [25,26,27]. The polymerized comonomers change the matrix crystallization because of the decline in the orderly arrangement of monomers in the polymer backbone [27,28]. Here, we refer to the concept of co-polyamide to partly destroy the structural regularity of the PA6 molecular main chain using the comonomers of hexamethylene adipamide salt at different copolymerization ratios, destroy and reform the hydrogen bond interaction between molecules during the spinning process, achieve a high tenacity up to 8.0 cN/dtex in the polyamide fiber preparation. The influence of the copolymerization ratio on the draw ability of the as-spun fibers, and the mechanical properties of the final high-tenacity fibers were systematically studied. The formation of hydrogen bonds between the carbonyl group and N–H, and the transition to crystal form for as-spun fibers under continuous heating were analyzed by in-site temperature-variable FTIR spectroscopy technology. The idea of achieving High-Strength PA6 fibers borrows the regulation of hydrogen bonds in the copolymerization of homologous polymer monomers, providing a new route for industrial mass production of high-performance fibers using a traditional synthetic polymer.

## 2. Materials and Methods

### 2.1. Raw Materials

Polyamide 6 (PA6) and co-polyamide 6/66s (CoPAs) with HA–salt ratios varying from 2% to 12% (by weight) were synthesized in a 10 L polymerization reactor according to our previous report [27,28]. Relative viscosities of all polyamides were controlled at 2.8 (at 25 °C and in a solvent of 96% concentrated sulfuric acid). For different CoPAs, the code of CoPA-x was used, where x means the HA–salt ratio in CoPAs.

In order to avoid the influence of impurities on the properties of the final fiber, all caprolactam residue in polymers was controlled below 0.1% and the moisture content was less than 0.02%. 

### 2.2. PA6 and CoPAs Fiber Preparation

**Figure 1:**
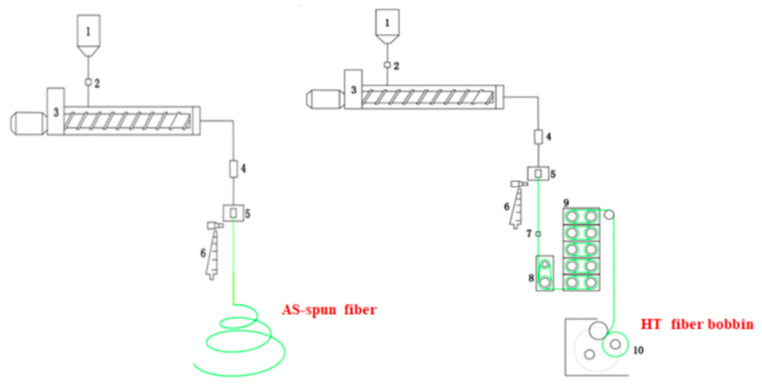
Schematic assembly of the high-tenacity polyamide fiber melt-spinning unit. 1. Hooper, 2. Feeding valve, 3. Extruder, 4. Gear bump, 5. Spinning pack, 6. Cooling system, 7. Oil bump, 8. GR1, 9. GR2, 10. Roller.

As the as-spun fiber (AS) and high-tenacity fiber (HT) of PA6 and CoPAs were produced using a Melton Spinning Equipment HY2 (Beijin Chonglee Machinery Engineering Co., Ltd., Beijing, China.) with specially designed draw units as shown in Figure 1. A single-screw extruder with a diameter of 25 mm and a length-to-diameter ratio (L/D) of 30 was used to melt and pressurize the polymers and the screw extrusion temperatures were graduated between 260 °C and 280 °C. A gear-metering pump was set to a nominal polymer mass flow rate of 45 g/min and a spin pressure of 160 bar was used. The molten polymer fluids were extruded through a 24-hole spinneret and then cooled in a cooling system with an air temperature of 20 °C, speed of 0.22–0.42 m/s, and relative humidity of 65–90%. A 5-stage draw unit was used to achieve the high-ratio draw and the total draw ratio (DR_max_) varied with the HA–salt ratio in the CoPAs, then a thermal heating unit was used to stabilize the condensed-state structure of the HTs and the highest thermal heating temperature was 195 °C, varying with the copolymerization ratio. Here, HT-x means the HT was prepared by CoPA-x. The final fineness of the HTs was 78D/24f and the as-spun fiber was free downstream of cooled melt fluid, cooling troughing the cooling system to the ground without oiling.

### 2.3. Measurement and Characterization

#### 2.3.1. Mechanical Properties

Tensile properties of HTs were measured on an automatic tensile tester USTER TENSORAPID 3 (Uster Technology CO., Ltd., Uster, Switzerland). First, a preload of 0.05 cN/dtex was applied to straighten the fiber. The starting length of the free fiber was 50.0 mm, and the crosshead speed was set at 380 mm/min. For each HT sample, the test was repeated 20 times, and the mean value and standard deviation were calculated. Before the test, all samples were conditioned at a room temperature of 20 ± 2 °C and relative humidity of 65% ± 2%. Coefficient of variance (CV) values were calculated according to standard ASTM D3822-07.

#### 2.3.2. In-Site Temperature-Variable FTIR Analysis

ATR-FTIR spectra were recorded with the spectrometer Vector 33-MIR (Bruker Optik, Ettlingen, Germany). The scan was scaled from 400 cm^−1^ to 4000 cm^−1^ under the working voltage of 220 to 240 V and the testing temperature evaluated from 60 to 220 °C in steps of 5 °C.

#### 2.3.3. X-ray Diffraction Analysis

X-ray diffraction analysis was fulfilled using an D8 Advance XRD (Bruker, Germany) with a Cu Kα radiation monochromatic filter in the 2θ range of 5°–50° and with a scan speed of 100 min^−1^.

#### 2.3.4. Thermal Properties

The molten and crystallization behavior of PA6 and CoPAs were characterized using differential scanning calorimetry (DSC) 214. (Netzsch, Selb, Germany) under an N_2_ atmosphere with a flow rate of 40 mL/min. First, the sample was heated from room temperature to 250 °C, followed by an isothermal step of 5 min to eliminate the thermal history, then a cooling ramp down to −40 °C was finished; after another isothermal step of 5 min at −40 °C, the second heating ramp up to 250 °C again was followed. Both heating and cooling rates were set to 10 °C/min.

#### 2.3.5. Two-Dimensional Spectroscopy Analysis

The 2DCS software was used for infrared two-dimensional spectra analysis [29,30]. The positive correlation intensity was shown by red areas in infrared two-dimensional spectra, whereas the negative correlation intensity was represented by blue areas.

## 3. Results

### 3.1. Drawn Properties of PA6 and CoPAs As-Spun Fibers

**Figure 2:**
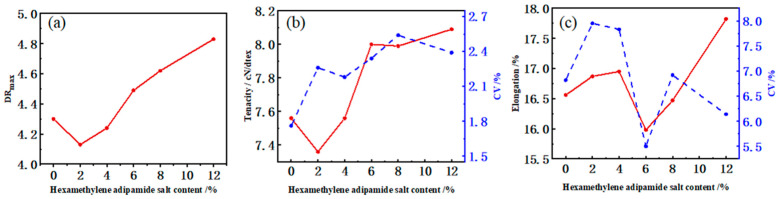
The DR_max_ (**a**) of PA6 and CoPAs as-spun fibers and mechanical properties (**b**,**c**) of PA6 and CoPAs HTs.

In order to obtain high-tenacity fibers, an as-spun fiber or fiber before the first draw stage characterized by high orientation and low crystallinity is first prepared, and then undergoes a high draw ratio (DR) process. Comparing the draw ability of PA6 and CoPAs as-spun fibers, the DR_max_ was investigated and the results are shown in Figure 2a. A linear increasing tendency was found for the DR_max_ value varying with the HA–salt ratio in CoPAs, which means the copolymerization favors the drawability of the polyamide chain. However, the lowest copolymerization ratio of 2% did not contribute to the modification because its DR_max_ was lower than that of PA6. In order to adjust the movement of the polyamide molecular chain during the draw process, a critical HA–salt ratio of CoPAs was needed.

The DR_max_ is a combined result of the crystallinity of the as-spun fiber, the physical entanglement state of the macromolecule chains, and the interaction between the chains [31]. In order to avoid the influence of the average molecular weight, the plasticization effect of water, and the residuals of monomers on the process and mechanical properties of the HTs, all spinning polymers were prepared with the same relative viscosity, as well as the caprolactam monomer residue and the moisture content. Thus, the influence of the DR_max_ is relative to the chemical structure of the polymer. The weak interaction between the polyamide chain is the efficient hydrogen bond interaction, while copolymerization of comonomers destroys the regularity of the macromolecule chain, changing the H-bonds’ density between macromolecular chains containing a functional group which can form hydrogen bonds [28]. PA66 monomer fixing inside the main chain of PA6 destroyed the regulation of the main chain, weakened the hydrogen bond interaction between adjacent molecules, and released the movement of the chain, which means deficient hydrogen bond formation provides a greater chance to orientate segments and lamellas in crystals during the drawing process in fiber formation. This tendency became evident with the increase in the HA–salt ratio in CoPAs until the HA–salt units inside the CoPAs can form a fixed hydrogen bond itself.

### 3.2. Mechanical Properties of PA6 and CoPAs High-Tenacity Fibers

Tenacity and elongation at the breaking point of PA6 and CoPAs HTs are shown in Figure 2b,c. The tenacity values show a minimum value at a HA–salt ratio of 2% for CoPA-2, a sharp levelling-up tendency was followed until the HA–salt ratio obtained 6%, and a slow growth trend was observed. However, the elongation at the breaking point showed a slight increase during the gradual change in the HA–salt ratio (variation was less than 2%). When the HA–salt ratio in CoPAs HTs was higher than 4%, the tenacity of CoPAs HTs is higher than PA6 HTs. A higher DR led to the higher tenacity of the fiber because of the higher orientation of molecular chains and crystalline lamella [32]; then, CoPAs can produce higher-tenacity fiber than PA6 when the HA–salt ratio is appropriate and effectively regulates the inter-molecular chains of the hydrogen bond.

According to the CV value of the CoPAs HTs, we found that a slightly increasing tendency of CV values were found for CoPAs tenacity value variation with HA–salt ratio, but the only evident increase tendency was found in elongation values variation at 2% and 4%, and then a reversing trend was found, which means copolymerization for high-tenacity PA6 fibers showed little influence on the uniformity of the mechanial properties of the fibers.

### 3.3. Crystallization Analysis of PA6 and CoPAs High-Tenacity Fibers

**Figure 3:**
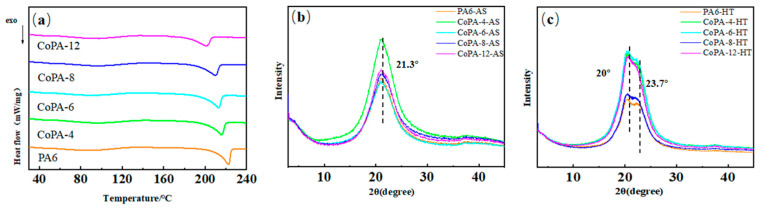
Thermal and crystallization performance analysis of PA6 and CoPAs from DSC (**a**) and Wide-angle X-ray diffraction scans for as-spun fibers (**b**) and high tenacity fibers (**c**).

Crystallization determined the mechanical properties of the fibers. In order to find the reason why CoPAs showed higher DR_max_ and tenacity than those of PA6, their thermal properties and crystallinities were studied, as shown in Figure 3, and detailed parameters are listed in Table 1. Considering the change tendency between CoPA-2 and CoPA-4 to be the same, only CoPA-4 was studied.

Based on the DSC curve, we found that copolymerization destroys the regular arrangement of the molecular chain, and thus decrease the melting point, as shown in Table 1; the tendency is increased with the HA–salt ratio in CoPAs. The peak melting temperature was 222.3 °C for PA6, while the value for CoPA-12 was 200.9 °C. With the increase of copolymerization ratio, the regularity of the molecular chain was destroyed and hindered the formation of PA6 crystal nuclei. Therefore, the melting temperature of CoPAs decreased gradually [27,33]. The onset melting temperature showed the same tendency, while the melting peak width became wider. The melt points plotted against the numbers of amide groups is the result of hydrogen bond interactions, which greatly increase the cohesive forces between the molecules [34]. The structure of polyamides with even numbers of CH_2_ groups are composed of sheets of fully extended planar chains, joined by hydrogen bonds, while the lower melting points of polyamides containing odd numbers of CH_2_ groups are the result of deficient hydrogen bond formation [34]. Both the hybrid main chain and the even and odd numbers of CH_2_ groups favored the decreased melting point of CoPAs.

The crystallinity of the fibers showed the same tendency, which means the PA6 had the highest crystallinity, and the CoPA-12 showed the lowest value based on DSC and XRD results. This tendency did not coincide with the mechanical test results that the high tenacity of the CoPA HT is not only the result of the crystallinity of the fiber, but another reason also dominated the featured performance.

Polyamide exhibits two common crystal structures of the γ crystalline form and α crystalline form depending on the stacking interaction between methylene groups [34]. For PA6, the α crystalline form is more stable than the γ crystalline form [35].

The as-spun fiber showed relatively high crystallinity for PA6, while decreasing sharply with the rise in the HA–salt ratio in CoPAs. That means the extensional flow on the spinning line did not favor the orientation-induced crystallization process for CoPAs, comparing with that of PA6. After the draw and thermal-setting step, the crystallinity of all HTs increased and PA6 obtained the highest value, while the CoPA-12, the lowest one. According to XRD curves, we found that peaks at 2θ ≈ 20 and 23.7°, marked two peaks of the α crystalline form of PA6 for HTs, while the peak at 21.3° identified the γ crystalline form of PA6 for AS samples [33].

For as-spun PA 6, the γ crystalline form has pseudohexagonal cells, and shorter chain repeat distances because of a favorable spatial arrangement of the amide groups [13,36]. For PA66, there are two different crystalline forms of α (2θ = 20.12 and 23.94°) and β (2θ = 21°) [37], while consisting chiefly of the α crystalline form. Two molecules form sheets of fully extended chains with the formation of hydrogen bonds. However, in our research, no evident α or β crystalline form was found for as-spun CoPA fibers.

### 3.4. Hydrogen Bond Interactions in Polyamide by Using In-Site Temperature-Variable FTIR Analysis

**Figure 4:**
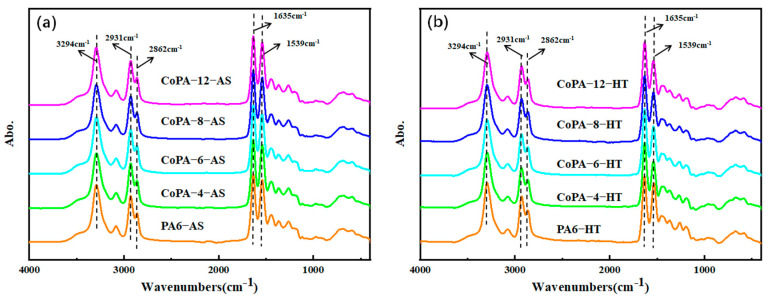
Typical attenuated in-site total reflection Fourier transform infrared spectra for as-spun (**a**) and HT (**b**) PA6 and CoPAs.

**Figure 5:**
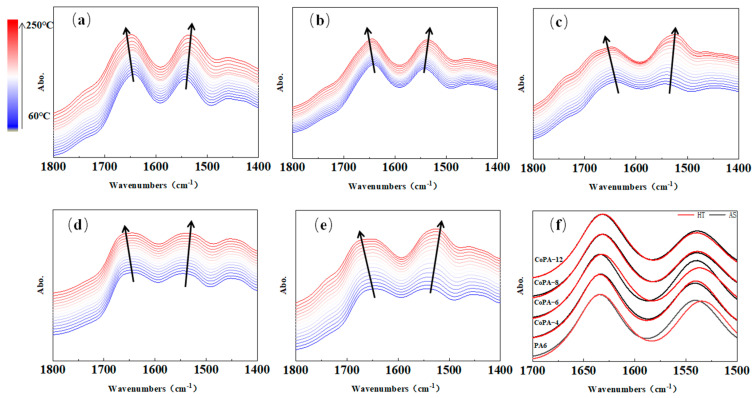
In-site temperature-variable FTIR spectra for polyamide 6 AS fibers (**a**), CoPA-x AS fibers with the x value of 4, 6, 8, and 12, respectively, under the wavenumber varying from 1800 cm^−1^ to 1400 cm^−1^ (**b**–**e**), and ATR-FTIR spectra for HTs and ASs of PA6 and CoPAs at 25 °C from 1700 cm^−1^ to 1500 cm^−1^ (**f**).

In-site temperature-variable FTIR analysis is a useful tool for the study of hydrogen bonds in polyamide [38,39] because the evident N–H stretching band, and the amide I and amide II band change in the infrared absorption spectra. Hydrogen bond interaction changes the shape and shifts the wave number of the bonds considerably compared with those of the non-bonded ones. The in-site temperature-variable FTIR analysis is illustrated in Figure 4, in which the 1635 cm^−1^ belongs to the C=O stretching vibration bond (Amide I mode), 1539 cm^−1^ is the N–H bending vibration bond (amide II mode), 3294 cm^−1^ is assigned to the hydrogen-bonded N–H stretching bond, and 2931 cm^−1^ and 2862 cm^−1^ are the anti-symmetric and symmetric CH_2_ stretching band of methylene groups, respectively [38]. The amide I bond is the characterization of the carbonyl group in polyamide and it has the tendency to form H-bonds with the N–H group. To carry out a detailed analysis of the special band change with the tested temperature, limited wave numbers in FTIR spectra were studied, as shown in Figure 5. For every analysis, CH_2_ stretching bands were marked as internal standard peak.

**Figure 6:**
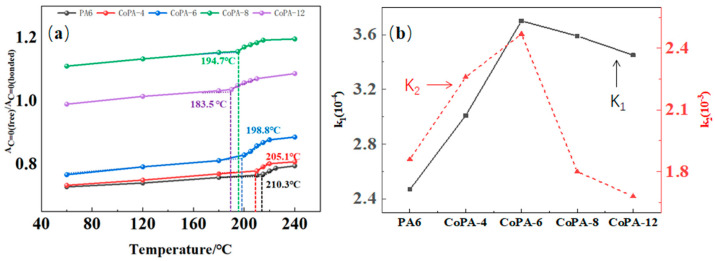
The temperature dependence of the fraction ratio of ordered hydrogen–bond and free carbonyl groups of PA6 and CoPAs AS fibers was calculated according to the area of fitted peak of free bond and bonded carbonyl groups (**a**), and the linear fit coefficients of the fraction ratio change tendency of before (K_1_) and after (K_2_) inflection points in curves of A_C=O free_/A_C=O bonded_ versus reciprocal temperature (**b**).

According to Figure 5, the amide I band is centered at 1635 cm^−1^ for as-spun fibers, but a widening trend could be found for CoPAs, especially for HA salt ratio more than 6%. Compared with the as-spun fiber, the characteristic peak of the HT fiber also changed (Figure 5f). The shoulder band at 1670 cm^−1^ on the left of 1643 cm^−1^ is multiplex peaks with the ordered (bonded hydrogen), and free C=O groups (unbonded hydrogen) [38], and it showed the conformational sensitization influenced by bonded hydrogen. According to the second-derivative spectra of amide I analysis, we detailed the area of the ordered and free C=O groups bands, and results were shown in Figure 6.

When the average molecular weight of polyamide is determined, the number of carbonyl groups in the molecular chain is fixed and the tendency of the carbonyl group forming hydrogen bond interactions with N–H is the result of the regular arrangement of adjacent molecular chains, the distance between molecular chains, and the movement of the molecular chain. Here, two factors provide the chance to change the formation tendency of hydrogen bond interaction for the carbonyl group, the elevated temperature and the disordered molecular regulation in polyamide because of the copolymerization. The PA66 comonomer is often introduced into the PA6 molecular chain to cleave the hydrogen bond by severing the molecular chain sequence of polyamide [40]. However, the function is always two-sided. Hydrogen bonds can limit the movement of the molecular chains; furthermore, they promote the crystallization of polyamide, at the same time promoting the mechanical properties of polyamide fibers [41]. 

From Figure 6, we can find that for all tested as-spun fibers, the chance to form bonded carbonyl groups decreased with the increase of tested temperature, and evident inflection points were found, which means the bonded carbonyl groups were evidently destroyed and formed free carbonyl groups. An increasing trend of PA6 and CoPAs was observed in Figure 6 and all points are relatively consistent with the onset melting temperature of CoPA HTs (Table 1), which means the hydrogen-bonded carbonyl group is destroyed when the main chain can move. As the scanning temperature increased, the bonds of the carbonyl group shifted to a higher wavenumber region. The movement of molecule segments and chemical bonds was accelerated, resulting in a decrease tendency in hydrogen bonds [6].

On the other hand, we found that the change tendencies were different with the HA–salt ratio in CoPAs, and both the linear fit coefficients of the fraction ratio change tendency of before (K1) and after (K2) inflection points in curves of A_C=O-free_/A_C=O-bonded_ versus reciprocal temperature showed the maximum value for CoPA-6, which means the bonded carbonyl group showed higher sensitivity to outside temperature than other CoPAs. On the other hand, the bonded carbonyl group inside CoPA-6 was more unstable. The maximum value means more than two factors influence the release of the bonded carbonyl group; here, in copolyamide, it is the regulation in the PA6 main chain destroyed by HA–salt comonomers and the possibility of PA66 forming stable structures by themselves.

In Figure 6 we also find that the ratio of bonded and free carbonyl groups showed different values. For PA6, and CoPA-4 and CoPA-6, the ratios were less than one under all tested temperature conditions. For CoPA-12 as-spun fibers, the ratio is less than one under lower temperatures, but for CoPA-8, the ratio was more than one at all tested temperature conditions. Considering the crystallinity of as-spun CoPA-8 fibers, a lot of free carbonyl groups must be exited in molecular chains. 

The amide I band shifts from 1635 cm^−1^ to 1637 cm^−1^ after the draw and thermal-setting treatment combined with the increase in the intensity, which improves the ordered region, increasing at the expense of the free and disordered regions. The orientation of the molecular chain provides a greater chance for regularly folded chains into the crystalline regions, favoring crystallization, and the thermal setting process fixes the state. The area ratio of fitted-peak free bonds and bonded carbonyl groups increased from 0.73 to 1.11 until the ratio obtained 8% at 60 °C. After that, it began to slowly decrease to 1.02.

**Figure 7:**
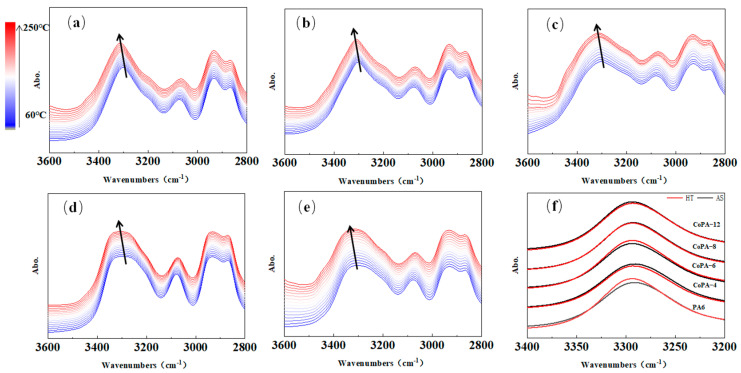
In-site temperature-variable FTIR spectra for polyamide 6 AS fibers (**a**), CoPA AS fibers under the wavenumber varying from 3600 cm^−1^ to 2800 cm^−1^ (**b**–**e**), and ATR-FTIR spectra for AS and HT of PA6 and CoPAs at 25 °C (**f**) from 3400 cm^−1^ to 3200 cm^−1^.

Since the carbonyl group forms hydrogen bond interactions with the N–H group in the polyamide main chain, we also discussed the N–H group using ATR-FTIR, as shown in Figure 7.

According to Figure 7, the N–H stretching bond for PA6, centered at 3294 cm^−1^ at room temperature, shifted to a higher wavenumber and became wider with increased temperature. Since the hydrogen-bonded N–H stretching band reflects both the crystalline phase and amorphous phase [13], different methods were used for the analysis of the special bonds. 

**Figure 8:**
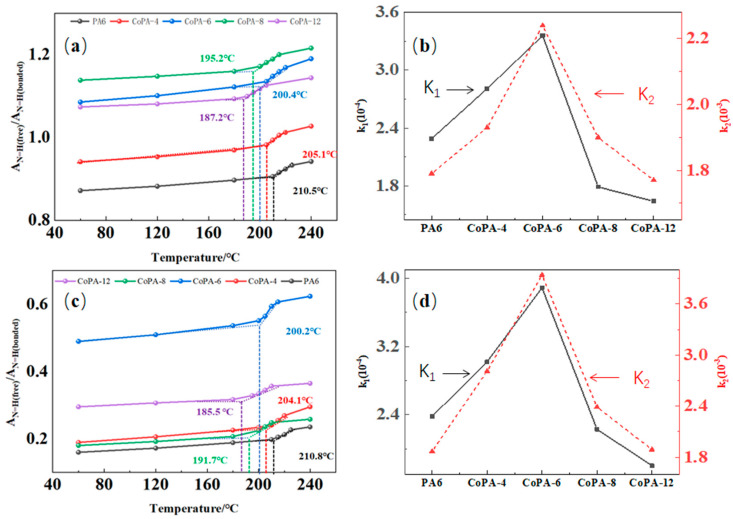
The temperature dependence of the fraction ratio of ordered hydrogen and free N–H bending vibration bonds (amide II mode) (1539 cm^−1^) (**a**) and the hydrogen-bonded N–H stretching bond (3294 cm^−1^) (**c**) of PA6 and CoPA AS fibers calculated according to the area of fitted-peak free bond and bonded N–H groups, and the linear fit coefficients of the fraction ratio change tendency of amide II mode (**b**) and the hydrogen-bonded N–H stretching bond (**d**) before (K1) and after (K2) inflection points in curves of A_N–H-free_/A_N–H-bonded_ versus reciprocal temperature.

According to Skrovanek’s method [38], the absorption coefficient of 3294 cm^−1^ depends strongly on the tested temperature and the strength of the hydrogen bond interactions, but according to Schroeder and cooper’s method [10], the absorption coefficient does not vary significantly with the temperature. For us, we only considered the absorption coefficient of bonded and free N–H groups, so the free N–H group band at 3480 cm^−1^ was considered. Remembering that water can also form hydrogen bond interactions with C=O in polyamide, and the typical bond is around 3200–3400 cm^−1^ in the spectrum, we also considered the N–H bending vibration bond (amide II mode), which was centered at 1539 cm^−1^ at room temperature; this bond belongs to bonded N–H [38]. The temperature dependence of the absorption coefficient characterized by the fraction ratio of ordered and free N–H bending vibration bonds at 1539 cm^−1^ and 3294 cm^−1^ for PA6 and CoPAs are shown in Figure 8a,c, and the linear fit coefficients of the two stages of Figure 8a,b are listed in Figure 8b,d.

From Figure 8, we found the same tendency as discussed before for carbonyl group: the bonded N–H group increased with the elevated temperature. The area ratio of free and bonded N–H bonds centered at 3294 cm^−1^ and 1539 cm^−1^ according to the second-derivative band in spectra, which showed the tendency of high-bonded N–H at 3294 cm^−1^ for all polyamide as-spun fibers (the ratio of A_N–H free_/A_N–H bonded_ less than 1), and bonded N–H was dominant only for PA6 and CoPA-4, which was the same tendency as discussed for the carbonyl group. The ratio of PA6 illustrated the lowest value, but the highest value belonged to CoPA-6 for the wavenumber centered at 3294 cm^−1^ and to CoPA-8 for the wavenumber centered at 1539 cm^−1^. The dependence of A_N–H free_/A_N–H bonded_ on temperature showed inflection points for all tested samples and all marked values are consistent with onset melting temperatures of CoPA HTs. The dependent slopes of A_N–H free_/A_N–H bonded_ on temperature curves before and after the inflection point are also shown as the maximum value for CoPA-6.

**Figure 9:**
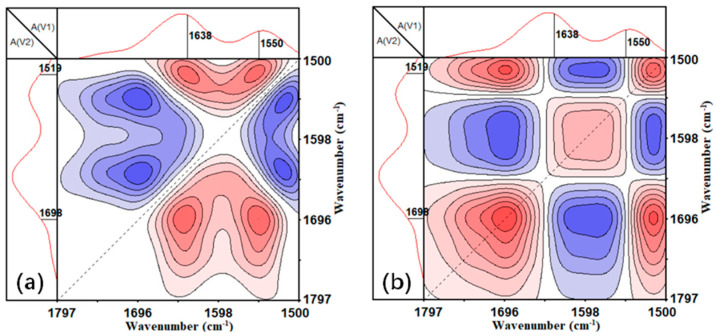
The synchronous (**a**) and asynchronous spectroscopy (**b**) of ATR-FTIR for CoPA-12 in a wavenumber range from 1500 to 1797 cm^−^^1^.

The total area changes of the N–H stretching region are induced not only by heat, but also by the tendency of hydrogen bonds between molecular chains, which are influenced by the composition of copolymerization. CoPA-6 showed the highest bonded–free transition for both the carbonyl group and N–H group because of the low forbidden groups inside molecules and it favors the movement of the main chain, which led to the high draw ratio of as-spun fibers and the higher tenacity of HT fibers. In order to analyze the response sequence between carbonyl group and N–H group, infrared two-dimensional spectroscopy analyses were used and the synchronous and asynchronous spectroscopy are shown in Figure 9.

It can be seen from the synchronous and asynchronous spectra that there was a correlation between carbonyl and N–H hydrogen bond destruction, and N–H moves before C=O, indicating that the possibility to form a less hydrogen-bonded state, and a higher tendency to stay as free groups and a less-bonded state for N–H group. 

The band of 3480 cm^−1^ may be associated with the O-H stretching vibrations in the bound water molecules in the amorphous region [34], which is very close to the free N–H band. For polyamide 66, no evident difference of the extenuation of hydrogen bond formation and no appreciable free NH absorption was observed at room temperature for drawn fibers, which means hydrogen bonds are completely formed between N–H and C=O groups in amorphous regions. However, for PA6 (odd number of CH_2_ groups) main chains, a perfect hydrogen bond interaction is more stable in the configuration than in the “trans” configuration. Thus, PA66 copolymerization comonomers distorted the regulation of PA6, but provided a greater chance of hydrogen bond formation when its ratio is high enough to create domains with a regular arrangement.

**Figure 10:**
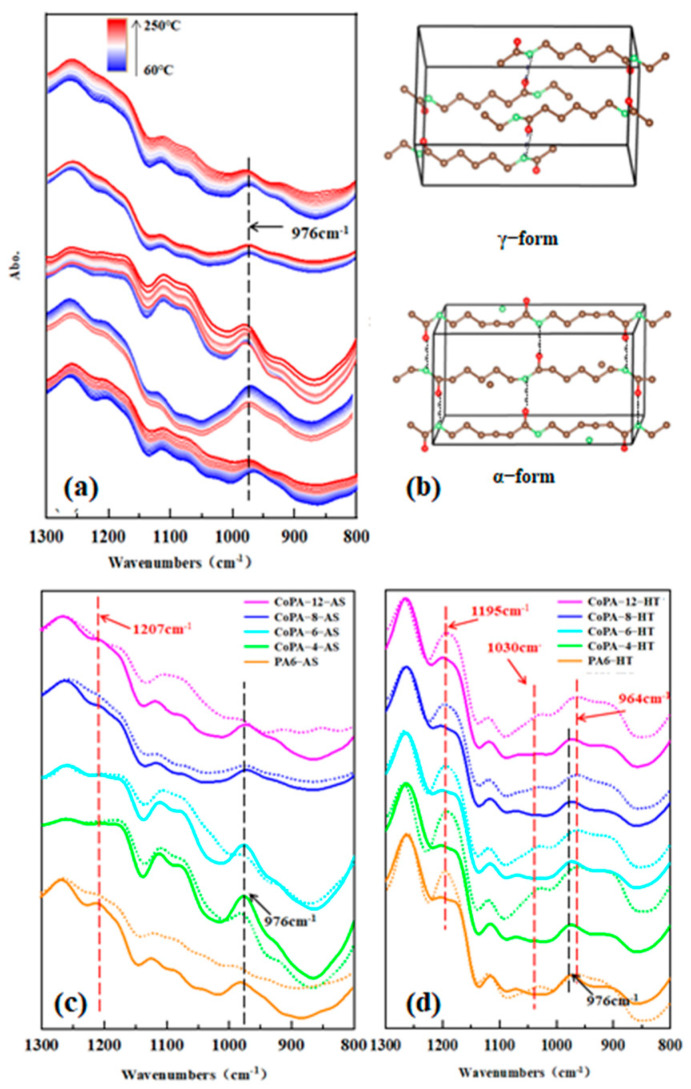
In-site ATR-FTIR spectra of PA6 and CoPAs AS fibers recorded from 60 °C (blue) to 220 °C (red) (**a**), at 60 °C (solid line) and 220 °C (dot line), the crystal structures of the α and γ crystalline forms (**b**) and AS fibers (**c**) vs. HT fibers (**d**) under wavenumbers varying from 1300 cm^−1^ to 800 cm^−1^.

Right now, the question is whether the hydrogen bond, which commonly exists in melted states, is stable in the crystalline state? In order to study the crystallization behavior of fibers, in-site temperature-variable FTIR analysis was also used for tracing the crystal form change with temperature and the draw and thermal-setting process, and results are illustrated in Figure 10.

In ATR-FTIR spectra for PA6 and CoPA HTs, the absorbance band at 976 cm^−1^ (black line) is representative of the γ crystalline form, while at 1195 cm^−1^ (shift to 1207 cm^−^^1^ for as-spun fiber), 1030 cm^−1^, and 964 cm^−1^ (red line) are the α crystalline form for PA6. For PA66, the absorbance band at 976 cm^−1^ is representative of the γ crystalline form and 933 cm^−1^ is representative of the α crystalline form [42,43]. Then, a hydrogen bond is formed between antiparallel chains in the α crystalline form, while being formed between twists in the γ crystalline form. Thus, the crystal density of the γ crystalline form is smaller than that of the α crystalline form, and the distance between adjacent amidic groups which formed hydrogen bonds is longer for the γ crystalline form than for the α crystalline form, which means interchain interactions in the γ crystalline form are smaller than the α crystalline form [36].

The results of temperature-variable ATR-FTIR noted that γ crystalline form absorbing bonds can be observed for PA6 and CoPA as-spun fibers, and that weak absorbing bonds for the α crystalline form when the tested temperature was higher than the melting point of the polyamide; the absorption bonds of the γ crystalline form became weak but did not completely disappear, and it is theoretically unreasonable because no crystal can be reserved in a molten state. Considering the test conditions, the tested sample was heated, programmed without a reserving period at testing temperature, and no shear force uploaded on the heated sample, but hydrogen bonds formed between localized molecular chains in the molten polymer still provided locally ordered structure, and the interchain interactions, such as hydrogen bonds, suppressed the shear slip of the crystalline chains during deformation [44].

For CoPAs and PA6, the vanished bonds of the γ crystalline form and the appeared bonds of the α crystalline form after the draw and thermal-setting process means more stable crystals formed after the process, and this is also mutually confirmed with the results of XRD (Table 1). Considering hydrogen bond interactions between the carbonyl group and N–H group, evidence for bonds for the α crystalline form in high-tenacity polymer fibers prove the hydrogen bonds are mostly in crystal, the hydrogen bond interactions between the molecular chain fixing the orientational segments.

## 4. Conclusions

In summary, higher-strength copolyamide 6/66 fibers were produced by adjusting the ratio of hexamethylene adipamide salt. The tensile strength of copolyamide increased to 8.0 cN/dtex when hexamethylene adipamide salt increased up to 6% and then changed smoothly. Based on the crystallization behavior analysis of polyamide 6 and copolyamide 6/66 high-tenacity fibers, the crystallinity of fibers showed a decreasing tendency, which suggested that the polyamide 6 had the highest crystallinity and the copolyamide 6/66 with the hexamethylene adipamide salt ratio of 12% showed the lowest value. In the XRD and FTIR analysis, the crystalline phase of as-spun fibers was mainly the γ crystalline phase, and the γ transformed into a more stable α crystalline form after drawing and a following thermal-setting process. 

The effect of hydrogen bond interactions between the N–H and C=O groups in adjacent chains on the tenacity of fibers was studied through temperature-variable FTIR. Infrared 2D spectroscopic analysis indicated that there is a correlation between the disruption of C=O and N–H hydrogen-bonding interactions, and the bonded N–H exhibits a faster tendency for hydrogen bonds to disrupt the group over bonded C=O. The copolymerization ratio had a direct effect on the formation–destruction of the hydrogen bond interactions between the C=O group and N–H group in the main chain of as-spun fibers, and the maximum change value was reached when the addition of hexamethylene adipamide salt was 6%, which was consistent with the inflection point of the final fiber strength change.

A stably oriented molecular segment structure in the macromolecular chain was formed by effective hydrogen bond interactions, which were fixed in the crystal structure. At the same time, the hydrogen bonds in the amorphous region promoted the orientation segment to form a local regular structure. Adjusting intermolecular hydrogen bonds through the copolymerization ratio permits the easy preparation of high-tenacity fibers.

## Figures and Tables

**Table 1:** Melting behavior and crystallization analysis of PA6 and CoPAs fibers.

Sample	DSC of HT					XRD of AS		XRD of HT	
	T_onset_ /°C	T_peak_ /°C	ΔT_m_ /°C	ΔH_m_ /J·g^−1^	*f_c_* /%	*f_c_* /%	Crystal Form	*f_c_* /%	Crystal Form
PA6	211.7	222.3	8.43	84.6	36.8	47.45	γ	67.6	α
CoPA-4	204.2	215.5	10.3	76.3	33.2	40.65	γ	61.2	α
CoPA-6	201.6	212.5	10.5	76.1	33.1	29.80	γ	56.7	α
CoPA-8	193.8	209.8	11.3	70.3	30.5	25.44	γ	46.2	α
CoPA-12	183.6	200.9	13.4	63.6	27.6	24.15	γ	41.9	α

Notes: 1 Tonset: The onset melting temperature; 2 Tpeak: The peak melting temperature; 3 ΔTm: The end melting temperature, minus the onset melting temperature; 4 ΔHm: The value of melting enthalpy; 5 *f*_c_: Degree of crystallinity.

## Data Availability

Data are contained within the article.
